# Integrative microbiome and functional profiling identify *Streptococcus anginosus* as a pro-tumorigenic driver in nasopharyngeal carcinoma

**DOI:** 10.1128/msystems.00357-26

**Published:** 2026-08-25

**Authors:** Yifan Zhu, Lewei Zhou, Chenyu Zhang, Zonglin Li, Tao Ding, Ying Su

**Affiliations:** 1 Department of Immunology and Microbiology, Zhongshan School of Medicine, Sun Yat-sen University 626117, Guangzhou, China; 2 Key Laboratory of Human Microbiome and Chronic Diseases (Sun Yat-sen University), Ministry of Education 26469, Guangzhou, China; The Forsyth Institute, Cambridge, Massachusetts, USA

**Keywords:** nasopharyngeal carcinoma, *Streptococcus anginosus*, host-microbe interaction, streptococcal TMPC, annexin A2, NF-κB signaling, peptide inhibitor

## Abstract

Oral microbial dysbiosis is associated with nasopharyngeal carcinoma (NPC), yet specific microbial drivers within this niche remain poorly characterized. Through multi-cohort profiling of saliva microbiota, we identified *Streptococcus* as consistently enriched in NPC, whose abundance positively correlates with advanced clinical stages and poor prognosis. Subsequent functional screening across multiple *Streptococcus* species revealed *S. anginosus* as the most potent promoter of NPC progression. At the molecular level, we established the interaction between the bacterial surface protein TMPC and host receptor ANXA2 as the primary signaling axis driving *S. anginosus*-induced tumorigenesis. Structural analysis revealed that specific conserved residues on TMPC confer *S. anginosus* with superior ANXA2-binding affinity compared with other streptococci, thereby triggering a robust NF-κB/TNFα-mediated pro-inflammatory program. Further underscoring its clinical relevance, high host ANXA2 expression significantly correlates with poorer overall survival in NPC patients. Targeted blockade of this interface using a competitive peptide effectively reduced intratumoral *S. anginosus* persistence and attenuated tumor progression *in vivo*. Collectively, our findings identify *S. anginosus* as a critical functional driver of NPC, with TMPC-ANXA2 acting as the pivotal molecular bridge coupling intratumoral persistence to a pro-tumorigenic inflammatory program. Targeting this specific host-microbe interface offers a promising non-antibiotic, precision therapeutic approach against microbiome-promoted NPC.

**IMPORTANCE:**

Nasopharyngeal carcinoma (NPC) has complex etiological links to microbial dysbiosis. Our study identifies *S. anginosus* as a key microbial driver, hijacking host ANXA2 via its surface protein TMPC to activate NF-κB/TNFα inflammatory signaling, promoting tumor progression. Structural insights into this interaction reveal targets for therapeutic intervention, with a competitive peptide showing efficacy in reducing bacterial persistence and tumor growth *in vivo*. This work redefines *S. anginosus* as a functional contributor to NPC, offering novel non-antibiotic strategies for targeting microbiota-driven cancers.

## INTRODUCTION

Nasopharyngeal carcinoma (NPC) is an aggressive epithelial malignancy with a distinct geographic distribution and a substantial global health burden (1–3). Despite advances in radiotherapy, chemotherapy, and immunotherapy, treatment resistance and disease recurrence remain common, highlighting the need to identify additional biological factors that contribute to NPC progression (2, 4–6).

Recent studies have increasingly implicated the microbiome as an active modulator of tumor biology, capable of shaping inflammatory tone, epithelial signaling, and therapeutic responsiveness (7–11). In NPC, microbial dysregulation has been reported, with increased bacterial burden and altered microbial composition observed in tumor tissues and adjacent mucosa compared with healthy individuals (12–16). Given the anatomical continuity between the oropharynx and nasopharynx, these observations suggest that the oral microbiome may serve as a primary reservoir influencing the nasopharyngeal oncogenic landscape (17–19).

Indeed, oral-to-nasopharyngeal microbial translocation has been documented in NPC (20, 21). Oral bacteria can be detected not only in the nasopharyngeal niche but also within the tumor tissues. Notably, higher levels of microbial translocation correlate with Epstein-Barr virus (EBV) reactivation, a central etiological driver of NPC (20). These findings suggest that oral microbiota may represent a previously underappreciated risk component shaping NPC development; however, the functional contribution of specific oral species to tumor progression and the molecular mechanisms governing these host-microbe interactions remain poorly understood.

In this study, we integrated microbiome profiling with functional assays, transcriptomic analyses, and *in vivo* tumor models to systematically investigate the role of oral microbes in NPC progression. We identify *Streptococcus anginosus* as a prominently enriched oral species that not only correlates with NPC progression but actively fuels tumor growth. We demonstrate that this bacterium drives sustained epithelial inflammatory programming through an interaction between the bacterial surface protein TMPC and the host receptor ANXA2, which subsequently activates the NF-κB signaling axis. These findings redefine *S. anginosus* from a passive bystander of dysbiosis to an active driver of NPC pathogenesis. Ultimately, our work highlights the TMPC-ANXA2 interaction as a promising therapeutic target, offering a viable approach to intercept tumor-promoting microbial crosstalk in NPC.

## RESULTS

### Oral *Streptococcus* is enriched and associated with disease progression in NPC

**Fig 1:**
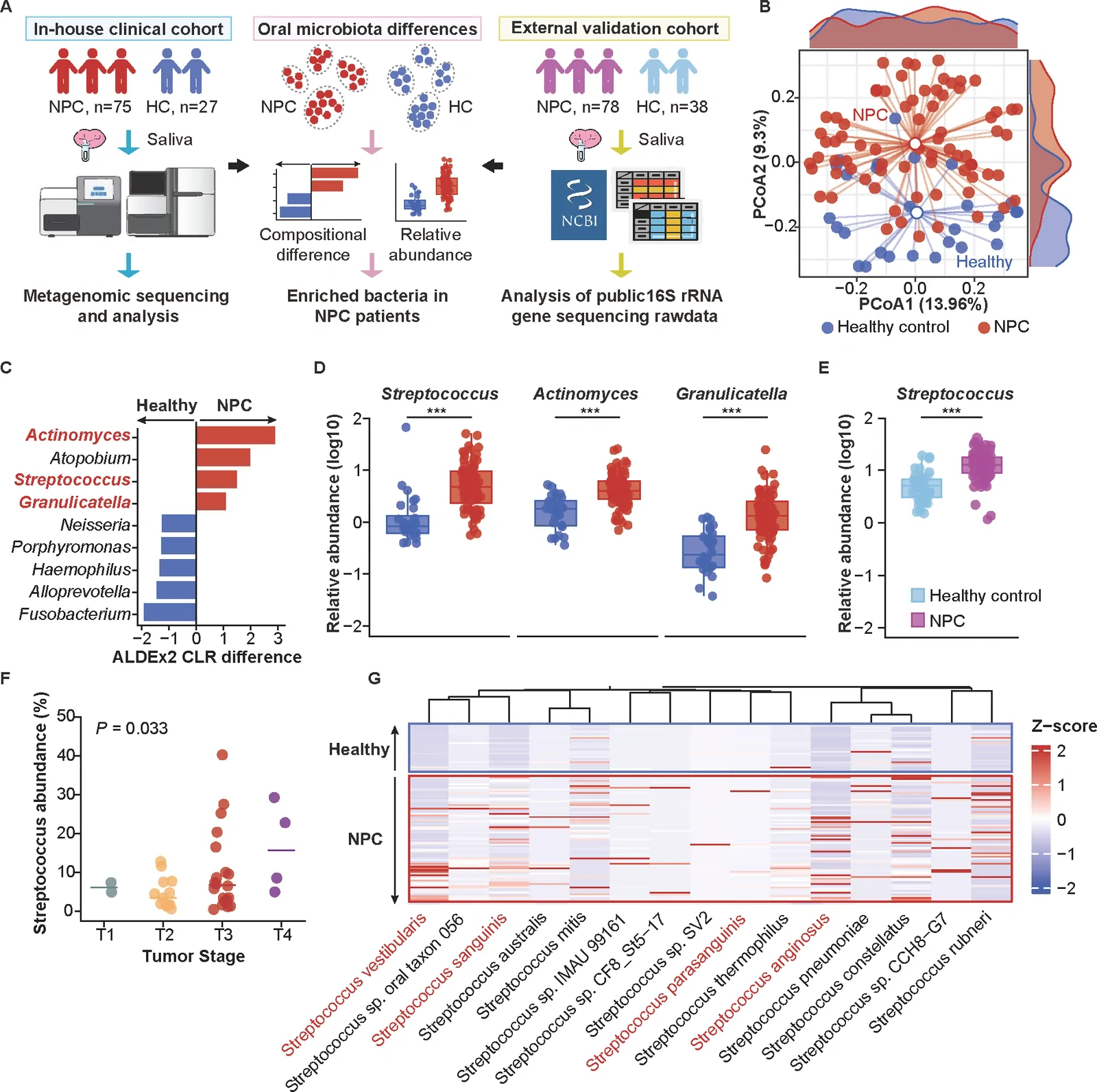
Oral *Streptococcus* is enriched and associated with disease progression in nasopharyngeal carcinoma (NPC). (**A**) Study design. An in-house discovery cohort (75 NPC patients and 27 healthy controls) was profiled using shotgun metagenomic sequencing of saliva. An independent external validation cohort (78 NPC patients and 38 healthy controls), with data generated by 16S rRNA gene sequencing, was used for validation. (**B**) Principal coordinate analysis (PCoA) based on Bray-Curtis dissimilarity showing the separation of oral microbial communities between NPC patients (*n* = 75) and healthy controls (*n* = 27) in the discovery cohort. Group differences were assessed using PERMANOVA (*R*² = 0.049, *P* = 0.001, 999 permutations). Homogeneity of multivariate dispersion was assessed using PERMDISP (*P* = 0.288), indicating no significant difference in within-group dispersion. (**C**) Differentially abundant bacterial genera between NPC patients (*n* = 75) and healthy controls (*n* = 27) identified by ALDEx2. Bars represent CLR differences, shown only for genera with absolute CLR difference >1. Genera highlighted in red (*Actinomyces*, *Streptococcus*, and *Granulicatella*) were consistently enriched in NPC across multiple differential-abundance methods, indicating robust associations. (**D**) Relative abundance of the three key genera across individual saliva samples from the discovery cohort. Relative abundances are shown on a log10-transformed scale. (**E**) Validation of *Streptococcus* enrichment in NPC using the external cohort (NPC, *n* = 78; controls, *n* = 38). Each point represents one biological sample. Relative abundances are shown on a log10-transformed scale. (**F**) Individual points represent patients, and horizontal lines indicate median values for each tumor stage. A monotonic increase in *Streptococcus* abundance across tumor stages (T1, *n* = 2; T2, *n* = 13; T3, *n* = 20; T4, *n* = 4) was tested using the Jonckheere-Terpstra trend test (two-sided, *P* = 0.033). (**G**) Species-level heatmap showing *Streptococcus* abundance across saliva samples in the discovery cohort. Values are Z-score scaled within each species. Species highlighted in red indicate taxa consistently enriched in NPC and isolated for downstream functional analyses. For box plots in panels **D** and **E**, boxes indicate the median and interquartile range, and whiskers represent 1.5× the interquartile range. Statistical significance was assessed using two-sided Wilcoxon rank-sum tests. *P < 0.05; **P < 0.01; ***P < 0.001; ns, not significant.

To characterize the oral microbial landscape in NPC, we performed shotgun metagenomic sequencing on a discovery cohort comprising 75 NPC patients and 27 age-matched healthy controls. To ensure the robustness, we further analyzed an independent external validation cohort from a previously published study, including 78 NPC patients and 38 healthy controls (Fig. 1A). Initial community-level comparisons revealed a distinct separation between NPC patients and healthy individuals (*R*² = 0.049, *P* = 0.001, 999 permutations), indicating a fundamental restructuring of the oral ecosystem in the context of NPC (Fig. 1B). Homogeneity of multivariate dispersion was not significant (PERMDISP, *P* = 0.288), suggesting that the observed separation reflects true compositional differences rather than increased variability within NPC samples.

To identify the taxa contributing to this shift, we applied multiple differential-abundance methods (ALDEx2, ANCOM, ANCOM-BC2, and LEfSe), which consistently highlighted *Actinomyces*, *Streptococcus*, and *Granulicatella* as enriched in NPC samples (Fig. 1C and Fig. S1A through C). Among these, *Streptococcus* exhibited the highest overall abundance in the discovery cohort (Fig. 1D). Examination of the external validation cohort confirmed that only *Streptococcus* showed a significant increase in NPC compared with healthy controls, whereas *Actinomyces* and *Granulicatella* did not differ significantly between groups (Fig. 1E and Fig. S1D). Collectively, these results establish *Streptococcus* as a robust signature genus associated with NPC.

The persistent enrichment of *Streptococcus* led us to question whether its expansion tracks with disease severity. By analyzing its distribution across different tumor stages, we observed that *Streptococcus* abundance positively correlated with advancing NPC stage, with higher levels in more advanced tumors (Fig. 1F). This clinical correlation suggests that oral *Streptococcus* may not be a mere bystander of dysbiosis, but rather a potential contributor to tumor aggressiveness.

Given that the *Streptococcus* genus comprises a diverse array of species with varied functional roles, we sought to refine our focus at the species level. Heatmap analysis of the most abundant streptococci revealed a heterogeneous enrichment pattern, where the genus-level expansion was primarily driven by a specific subset of species. Notably, species, such as *S. anginosus*, *Streptococcus sanguinis*, *Streptococcus parasanguinis*, and *Streptococcus vestibularis*, were consistently enriched across NPC samples (Fig. 1G).

To further examine whether streptococcal signals could also be detected in the nasopharyngeal tumor niche, we reanalyzed publicly available metagenomic sequencing data from NPC tumor tissues generated from the previous study (12). This analysis detected multiple *Streptococcus* species, such as *S. anginosus* and *S. sanguinis*, among the bacterial species present in tumor tissue-derived metagenomic profiles (Fig. S1E). This result supports the presence of *Streptococcus* signals within NPC tumor tissues and strengthens the biological link between salivary enrichment and the nasopharyngeal tumor microenvironment. Together, these findings narrowed the scope of our investigation to a group of NPC-associated streptococci and provided a rational basis for selecting candidates for downstream functional investigation.

### *S. anginosus* is the most potent pro-tumor *Streptococcus* species *in vitro* and *in vivo*

**Fig 2:**
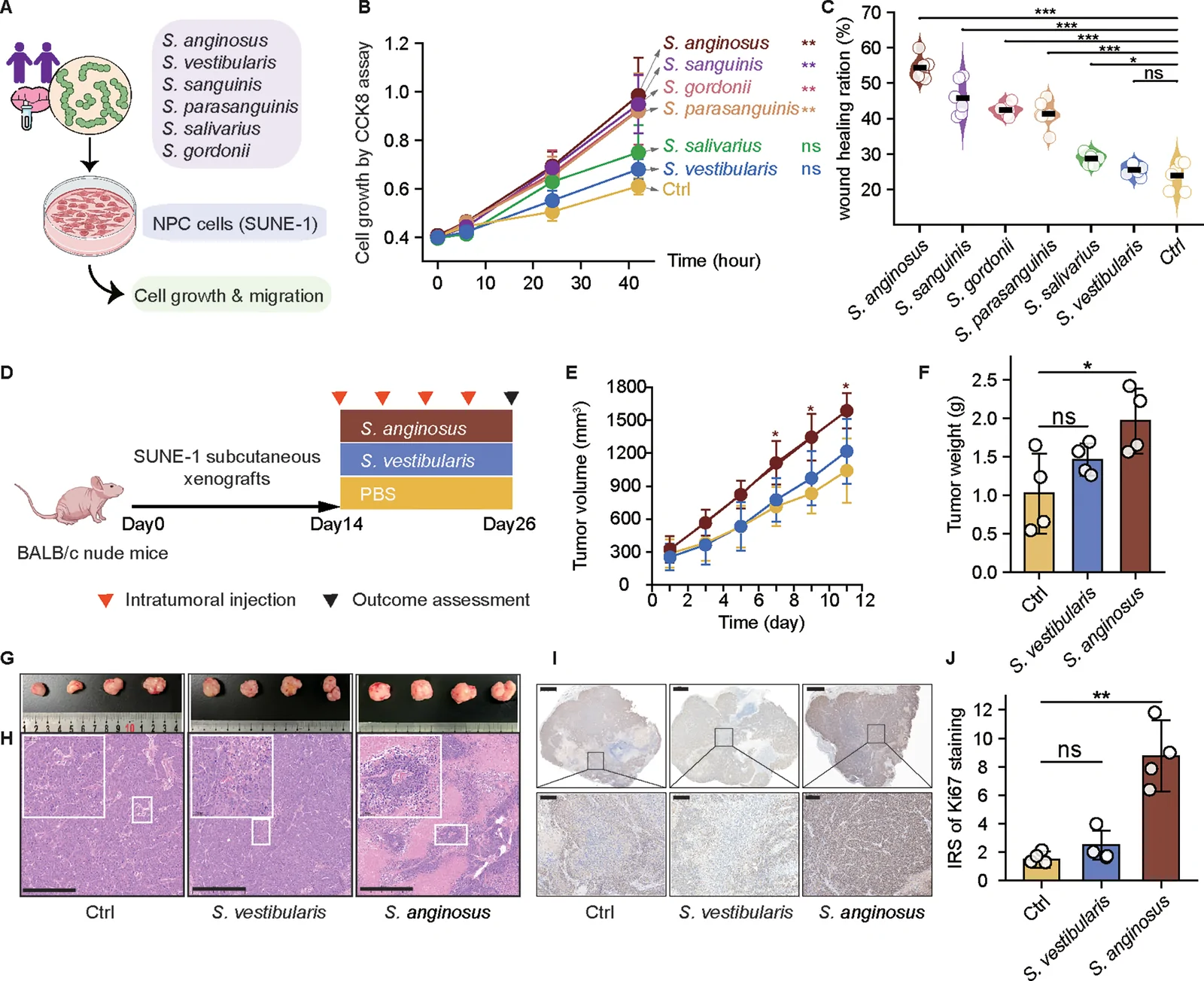
*S. anginosus* is the most potent pro-tumor *Streptococcus* species *in vitro* and *in vivo*. (**A**) Schematic overview of the *in vitro* functional screening strategy using SUNE-1 nasopharyngeal carcinoma cells co-cultured with representative oral *Streptococcus* species. (**B**) SUNE-1 cell proliferation following co-culture with individual *Streptococcus* species, measured by CCK-8 assay at the indicated time points (*n* = 6). (**C**) Quantification of wound closure in scratch-wound assays after co-culture with individual *Streptococcus* species (*n* = 6). (**D**) Experimental design of the subcutaneous SUNE-1 xenograft model with intratumoral administration of *S. anginosus*, *S. vestibularis*, or PBS control. (**E**) Tumor growth curves of SUNE-1 xenografts under the indicated treatment conditions (*n* = 4 mice per group). (**F**) Tumor weights measured at the experimental endpoint (*n* = 4 mice per group). (**G**) Representative images of excised tumors from each treatment group. (**H**) Representative hematoxylin and eosin (H&E)-stained tumor sections from each group (*n* = 4 mice per group). Scale bars, 500 μm. (**I**) Representative Ki67 immunohistochemical staining of tumor sections. (Scale bars, 1,000 μm [top] and 200 μm [bottom].) (**J**) Corresponding quantification of Ki67 immunoreactive scores (IRS) (*n* = 4 mice per group). Data are presented as mean ± SD. Statistical significance was determined using one-way ANOVA followed by Tukey’s HSD test (**C, F, and J**) or the Kruskal-Wallis test, followed by Dunn’s multiple comparisons test (**B, E**). *P < 0.05; **P < 0.01; ***P < 0.001; ns, not significant.

To evaluate the functional relevance of *Streptococcus* species enriched in NPC, we performed a co-culture screening using six representative species isolated from the saliva of NPC patients (Fig. 2A). We observed varying degrees of pro-tumorigenic potential among these species. While *S. anginosus, S. sanguinis, S. gordonii*, and *S. parasanguinis* significantly promoted the proliferation of SUNE-1 cells, *S. anginosus* induced the most sustained proliferative response (Fig. 2B and Fig. S2A). In contrast, *S. salivarius* and *S. vestibularis* exhibited minimal effects on cell growth.

Migration assays showed a consistent trend, with *S. anginosus* accelerating wound closure, whereas *S. vestibularis* showed negligible impact on cell motility (Fig. 2C and Fig. S2B). These *in vitro* findings identified *S. anginosus* as the most potent pro-tumor candidate and suggested *S. vestibularis* as a suitable low-activity control for further validation.

Guided by these divergent functional profiles, we sought to validate our findings *in vivo*. We established a subcutaneous NPC xenograft model, employing *S. anginosus* as the putative pathogen and *S. vestibularis* as a bacterial control (Fig. 2D). To verify local bacterial persistence in tumor tissues, we performed species-specific qPCR after intratumoral administration. Both *S. anginosus* and *S. vestibularis* were detectable in their corresponding treatment groups, whereas only limited signals were detected in the mock-treated controls, confirming successful local bacterial presence within tumor tissues (Fig. S3D). The *in vivo* results were consistent with our cellular observations. *S. anginosus* administration significantly accelerated tumor growth and increased final tumor weight, whereas tumors treated with *S. vestibularis* exhibited growth patterns comparable to the mock-treated group (Fig. 2E through G). Histopathological evaluation showed that *S. anginosus*-treated tumors developed a more aggressive morphology, characterized by increased cellular density and reduced tissue organization (Fig. 2H). This was further supported by Ki67 immunohistochemistry, which revealed a markedly elevated proliferative index in the *S. anginosus* group (Fig. 2I and J). Together, these results identify *S. anginosus* as a key driver of NPC progression among the enriched streptococci, providing the basis for further mechanistic investigation.

### *S. anginosus* drives a pro-inflammatory transcriptional program in NPC

**Fig 3:**
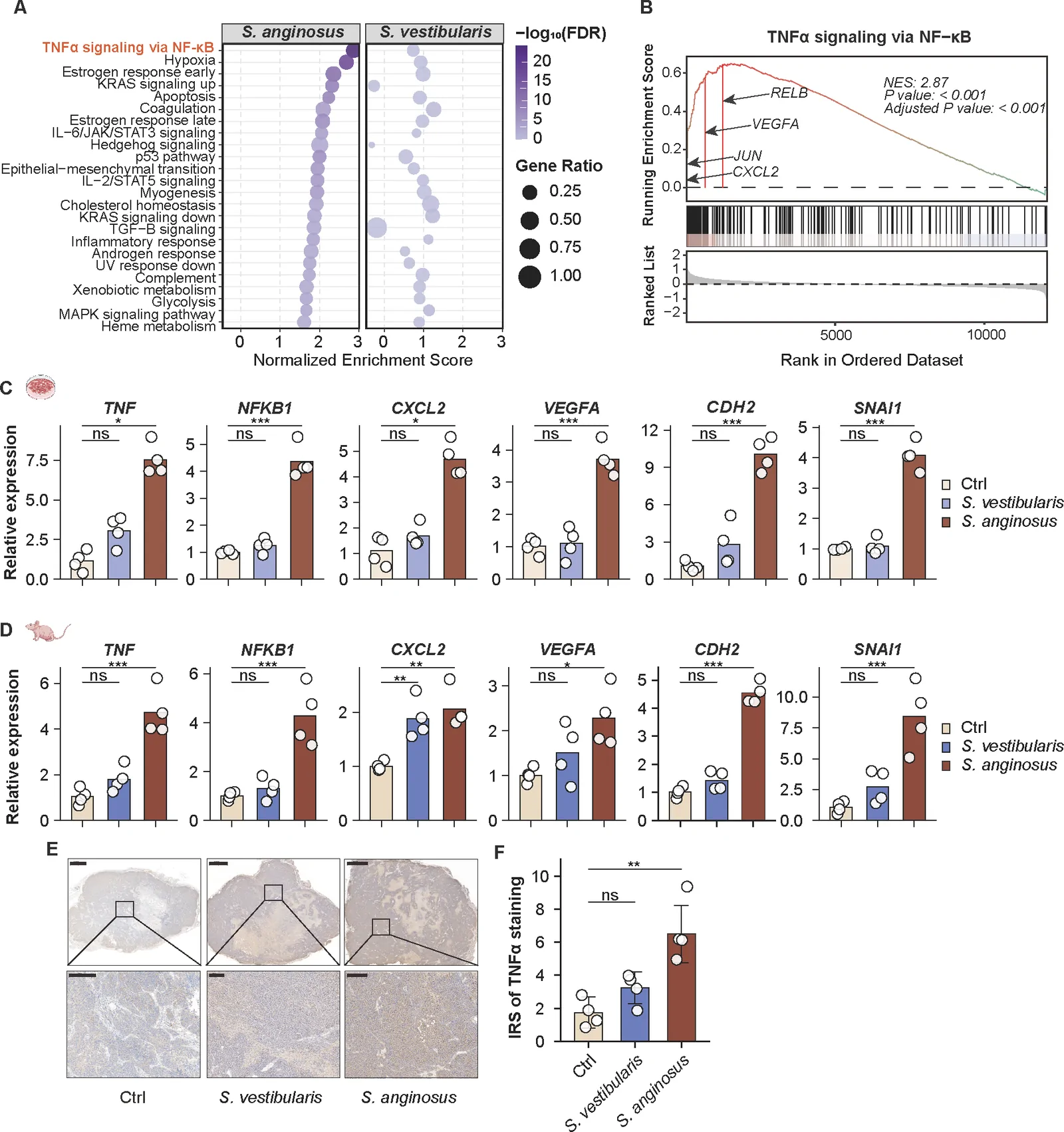
*S. anginosus* induces a species-dependent pro-inflammatory transcriptional program in NPC cells and tumors. (**A**) Pathway enrichment analysis of RNA sequencing data from SUNE-1 cells exposed to *S. anginosus* or *S. vestibularis* (*n* = 3 per condition). Dot plots display significantly enriched pathways ranked by normalized enrichment score (NES); dot size indicates gene ratio and color denotes −log_10_ (FDR). Selected hallmark and inflammation-related pathways are shown. (**B**) Gene set enrichment analysis (GSEA) of the TNFα signaling via NF-κB pathway in SUNE-1 cells following *S. anginosus* exposure (*n* = 3 per condition). The running enrichment score across the ranked gene list is shown; vertical bars indicate the positions of pathway genes. Representative leading-edge genes are annotated. (**C**) Quantitative PCR analysis of NF-κB-associated inflammatory genes (*TNF*, *NFKB1*, *CXCL2,* and *VEGFA*) and epithelial-mesenchymal transition (EMT) markers (*CDH2* and *SNAI1*) in SUNE-1 cells treated with *S. anginosus* or *S. vestibularis* (*n* = 4 per group). Expression levels are shown relative to control cells. (**D**) Quantitative PCR analysis of the same NF-κB-associated inflammatory genes and EMT markers in xenograft tumor tissues following exposure to *S. anginosus* or *S. vestibularis* (*n* = 4 per group). Expression levels are shown relative to control tumors. (**E**) Representative immunohistochemical staining of TNFα in xenograft tumors from control, *S. vestibularis*-treated, and *S. anginosus*-treated groups (top, low magnification; bottom, higher magnification; *n* = 4 tumors per group). Scale bars, 1000 μm (top) and 200 μm (bottom). (**F**) Corresponding quantification of TNFα staining based on immunoreactivity score (IRS). For **C, D, and F**, data are presented as mean ± SD. Statistical significance was determined using Welch’s ANOVA followed by the Games-Howell *post hoc* test (**C, D**) or one-way ANOVA followed by Tukey’s HSD test (**F**). *P < 0.05; **P < 0.01; ***P < 0.001; ns, not significant.

To delineate the molecular alterations underlying the pro-tumorigenic effects of *S. anginosus*, we performed transcriptomic profiling of SUNE-1 cells. This analysis revealed a species-dependent activation of inflammatory and oncogenic transcriptional programs, with *S. anginosus* inducing stronger inflammatory and oncogenic signatures than the control oral streptococcal species. Pathway enrichment analysis identified TNFα signaling via NF-κB as the most prominently activated cascade in *S. anginosus*-treated cells, exhibiting the highest normalized enrichment score (NES) and statistical significance (Fig. 3A). Additional inflammation-associated pathways, including IL-6/JAK/STAT3 signaling and the general inflammatory response, were also significantly enriched. Importantly, cancer hallmark pathways, such as hypoxia, epithelial-mesenchymal transition (EMT), and KRAS signaling were similarly activated. In contrast, *S. vestibularis* failed to induce comparable pathway activation (Fig. 3A).

Gene set enrichment analysis (GSEA) further confirmed strong activation of the TNFα/NF-κB axis in *S. anginosus*-treated cells (NES = 2.87, *P* < 0.001; Fig. 3B), driven by NF-κB-associated genes, such as *RELB*, *CXCL2,* and *JUN*. Genes involved in angiogenesis and survival signaling, including *VEGFA,* were also upregulated. Conversely, *S. vestibularis* showed no enrichment of TNFα/NF-κB signaling (NES = 0.74), supporting a species-dependent effect of *S. anginosus* relative to *S. vestibularis* (Fig. S3A). Quantitative PCR analysis validated these findings, showing that exposure to *S. anginosus* significantly increased the expression of NF-κB-associated inflammatory genes (including *TNF, NFKB1, CXCL2, VEGFA, RELB,* and *JUN*), while *S. vestibularis* induced no significant change (Fig. 3C and Fig. S3B). Additionally, mesenchymal markers (*CDH2*, *SNAI1*, and *VIM*) were elevated following *S. anginosus* exposure, whereas their expression remained unchanged in the *S. vestibularis* group (Fig. 3C and Fig. S3B).

To determine whether this transcriptional signature is maintained *in vivo,* we evaluated gene expression in our xenograft tissues. Tumors exposed to *S. anginosus* displayed consistent upregulation of NF-κB-associated inflammatory genes and EMT markers, whereas tumors treated with *S. vestibularis* showed minimal changes (Fig. 3D and Fig. S3C). Given the central position of TNFα within the enriched NF-κB signaling network, we assessed its protein-level expression by immunohistochemistry. TNFα staining intensity was significantly increased in tumors from the *S. anginosus* group, while minimal staining was observed in the control and *S. vestibularis*-treated tumors (Fig. 3E and F). Together, these results establish *S. anginosus* as a pro-inflammatory driver of NPC progression, characterized by a specific transcriptional signature that highlights its role in modulating NPC biology.

### TMPC-ANXA2 interaction dictates *S. anginosus*-driven malignant phenotypes in NPC

**Fig 4:**
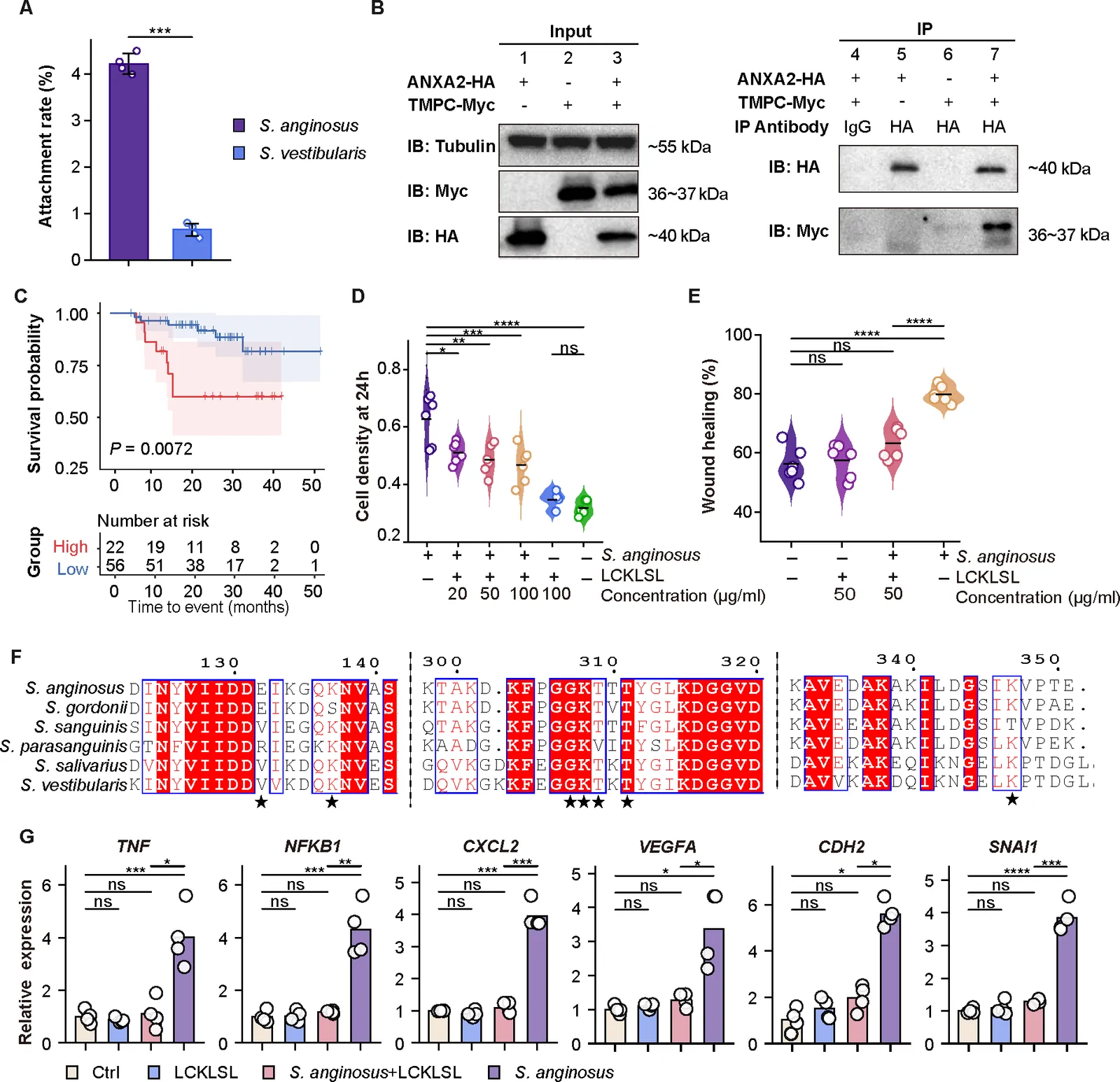
The TMPC-ANXA2 interaction mediates *S. anginosus*-driven malignant phenotypes in NPC. (**A**) Quantification of bacterial adhesion to SUNE-1 cells following a 2-h co-incubation with *S. anginosus* or *S. vestibularis* (*n* = 4 per group). Adherent bacteria were quantified by colony-forming unit (CFU) assays after removal of non-adherent bacteria. (**B**) Co-immunoprecipitation (Co-IP) analysis of TMPC and ANXA2 interaction in HEK293T cells. Whole-cell lysates (lanes 1–3, Input) were analyzed by Western blot to confirm the expression of ANXA2-HA and TMPC-Myc. For Co-IP assays, cell lysates were immunoprecipitated using anti-HA antibody, with IgG serving as a negative control (lane 4). The immunoprecipitated complexes were analyzed by Western blot using anti-HA and anti-Myc antibodies (lanes 5–7). (**C**) Kaplan-Meier analysis of overall survival in NPC patients from the GSE102349 cohort (*n* = 78), stratified by tumoral ANXA2 expression. High ANXA2 expression is associated with significantly shorter overall survival (*P* = 0.0072, log-rank test). (**D**) Effects of pharmacological disruption of the TMPC-ANXA2 interaction on SUNE-1 cell proliferation. Cells were treated with the ANXA2-targeting competitive peptide inhibitor LCKLSL at the indicated concentrations in the presence or absence of *S. anginosus* (*n* = 6 per condition). Cell proliferation was assessed by CCK-8 assay. (**E**) Quantification of wound closure in scratch assays following co-culture with *S. anginosus* in the presence or absence of LCKLSL (*n* = 6 per condition). (**F**) Multiple sequence alignment of TMPC homologs from six oral *Streptococcus* species. Residues corresponding to the predicted ANXA2-binding interface are indicated by asterisks, highlighting conservation in *S. anginosus* and divergence in weakly active species. (**G**) Quantitative PCR analysis of NF-κB-associated inflammatory genes and EMT markers in SUNE-1 cells treated as indicated (*n* = 4 per group). For panels **A, D, and E,** and** G**, data are presented as mean ± SD. Statistical significance was determined using two-sided Student’s *t*-tests (**A**), one-way ANOVA followed by Tukey’s HSD test (**D, E**), or Welch’s ANOVA followed by the Games-Howell *post-hoc* test (**G**). *P < 0.05; **P < 0.01; ***P < 0.001; ****P < 0.0001; ns, not significant.

Bacterial adhesion to host cells is the critical first step in microbe-tumor interactions. To determine if the pro-malignant traits of *S. anginosus* correlate with its capacity for host cell engagement, we compared its attachment to SUNE-1 cells with that of the weakly active *S. vestibularis*. Colony-forming assays revealed a significantly higher adhesion rate for *S. anginosus* (Fig. 4A), indicating that efficient physical attachment is a prerequisite for its oncogenic effect.

Previous studies have identified the streptococcal surface protein TMPC and the host membrane protein annexin A2 (ANXA2) as a crucial adhesion module in other epithelial models. We therefore hypothesized that *S. anginosus* exploits this specific axis to anchor onto NPC cells. To test this, we first investigated the clinical relevance of ANXA2 expression in NPC tumor tissues. Supporting the clinical significance of this host target, Kaplan-Meier survival analysis of NPC cohorts revealed that high tumoral ANXA2 expression is significantly associated with poorer overall survival (Fig. 4C; *P* = 0.0072). This suggests that elevated ANXA2 abundance may create a permissive niche for *S. anginosus* persistence, thereby accelerating disease progression.

In addition, co-immunoprecipitation (Co-IP) analysis provided direct biochemical evidence supporting the interaction between TMPC and ANXA2 in a heterologous expression system (Fig. 4B). In HEK293T cells co-expressing tagged TMPC and ANXA2, TMPC was specifically detected in the ANXA2 immunocomplex following anti-HA immunoprecipitation, whereas no corresponding signal was observed in IgG or single-transfection controls, confirming that TMPC and ANXA2 form a protein complex in cells.

To elucidate the molecular basis of this binding, we performed structural modeling using AlphaFold 3. The prediction revealed a stable TMPC-ANXA2 complex driven by a discrete interface enriched with complementary charged and polar residues, such as the interactions between TMPC (e.g., Glu132, Lys137, Gly307) and ANXA2 (e.g., Asp187, Glu189) (Fig. S4A). Furthermore, multiple sequence alignment across oral streptococci demonstrated that *S. anginosus* strictly conserves these critical ANXA2-binding residues (Fig. 4F). In contrast, *S. vestibularis* exhibits multiple amino acid substitutions at these key sites, which may explain its impaired capacity for host cell attachment.

Finally, to definitively prove that TMPC-ANXA2 engagement is functionally required to trigger the malignant phenotypes, we disrupted this physical interface using LCKLSL, a specific competitive peptide inhibitor. Blockade of ANXA2 significantly abrogated *S. anginosus*-induced NPC cell proliferation in a dose-dependent manner (Fig. 4D), without affecting intrinsic bacterial viability (Fig. S4D). Crucially, LCKLSL treatment also effectively suppressed the *S. anginosus*-driven cell migration (Fig. 4E and Fig. S4E) and completely reversed the induction of the core NF-κB inflammatory and EMT gene signature (Fig. 4G and Fig. S4F). Collectively, these data demonstrate that *S. anginosus* hijacks the host ANXA2 receptor via its surface TMPC protein to initiate downstream pro-inflammatory and pro-malignant signaling.

### Disruption of the TMPC-ANXA2 interaction restrains tumor progression *in vivo*

**Fig 5:**
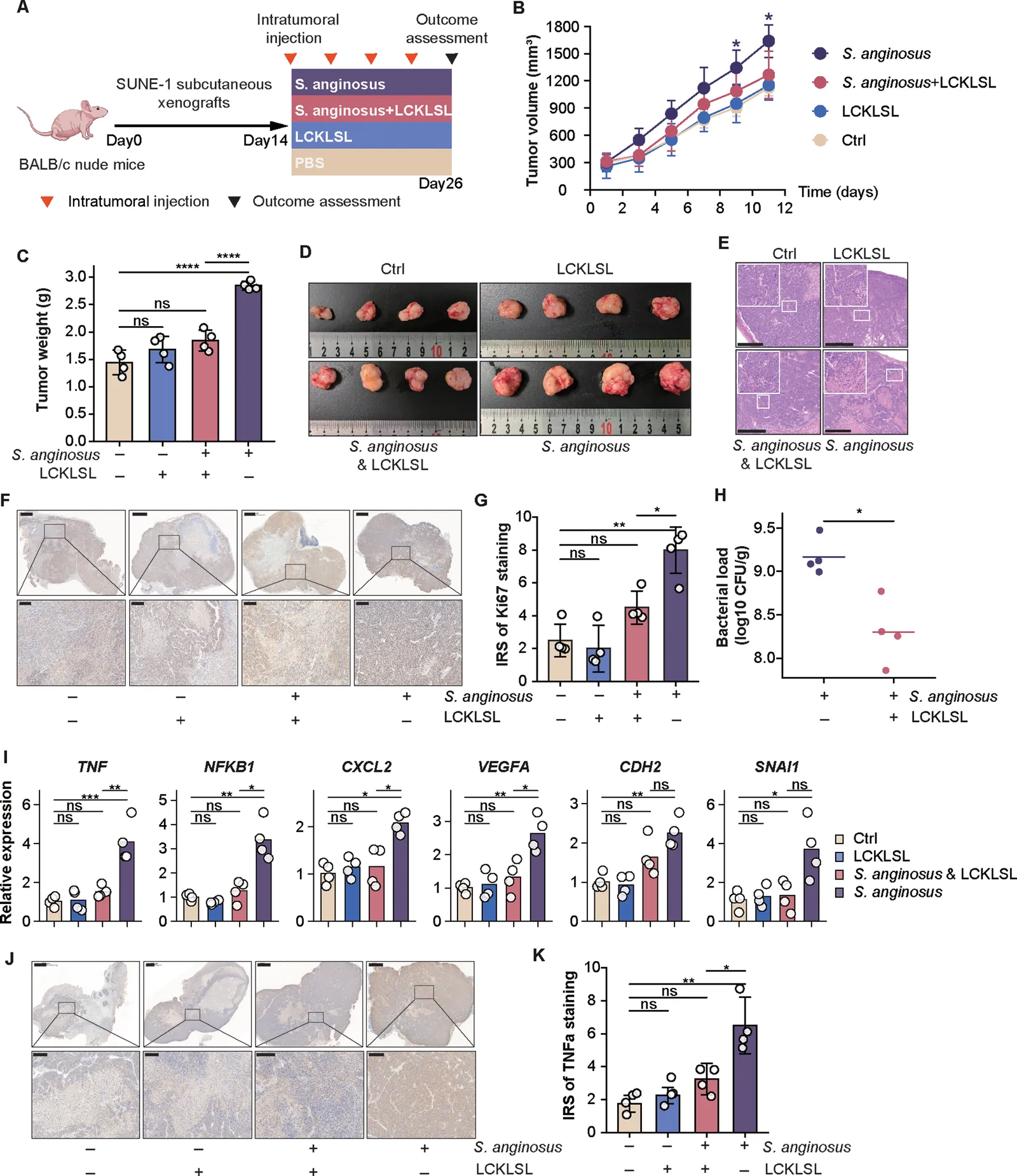
Disruption of the TMPC-ANXA2 interaction restrains *S. anginosus*-driven tumor progression *in vivo*. (**A**) Schematic illustration of the subcutaneous NPC xenograft model and treatment regimen (*n* = 4 mice per group). (**B**) Tumor growth curves of SUNE-1 xenografts in BALB/c nude mice under the indicated treatment conditions. (**C**) Final tumor weights at the experimental endpoint (day 26). (**D**) Representative images of excised xenograft tumors from each group. (**E**) Representative hematoxylin and eosin (H&E)-stained tumor sections. Scale bars, 500 μm. (**F**) Representative immunohistochemical (IHC) staining of Ki67 in xenograft tumor sections. Scale bars, 1000 μm (top) and 200 μm (bottom). (**G**) Quantification of Ki67 staining based on immunoreactivity scores (IRS). (**H**) Intratumoral bacterial burden quantified as log_10_ CFU per gram of tumor tissue. (**I**) Quantitative PCR analysis of NF-κB-associated inflammatory genes (*TNF*, *NFKB1*, *CXCL2*, and *VEGFA*) and EMT-related markers (*CDH2* and *SNAI1*) in tumor tissues. (**J**) Representative IHC staining of TNFα in tumor sections. Scale bars, 1,000 μm (top) and 200 μm (bottom). (**K**) Corresponding quantification of TNFα immunoreactivity scores (IRS). Data are presented as mean ± SD. Statistical significance was determined using one-way ANOVA, followed by Tukey’s HSD test (**C, G, and K**), two-sided Student’s *t*-tests (**H**), Welch’s ANOVA, followed by the Games-Howell *post-hoc* test (**I**), or the Kruskal-Wallis test, followed by Dunn’s test (**B**). *P < 0.05; **P < 0.01; ***P < 0.001; ****P < 0.0001; ns, not significant.

Given the requisite role of TMPC-ANXA2 engagement in driving *S. anginosus*-induced malignant phenotypes *in vitro*, we next evaluated its therapeutic potential using a subcutaneous NPC xenograft model. Tumor-bearing mice were treated with *S. anginosus*, the specific peptide inhibitor LCKLSL, or their combination, alongside untreated controls (Fig. 5A). While administration of LCKLSL alone had no baseline effect on tumor growth, co-treatment with LCKLSL markedly abrogated the accelerated tumor progression induced by *S. anginosus*. This resulted in significantly restricted tumor growth kinetics and substantially lower final tumor weights (Fig. 5B through D).

Histopathological evaluation (H&E) confirmed a profound reduction in tumor aggressiveness upon disruption of this host-microbe interface. Tumors exposed to *S. anginosus* alone displayed hallmark features of high malignancy, including hypercellularity, severe loss of tissue architecture, extensive necrosis, and prominent microvascular proliferation. Importantly, these aggressive pathologies were substantially reversed by LCKLSL co-treatment (Fig. 5E). Consistently, Ki67 immunohistochemistry revealed a pronounced suppression of *S. anginosus*-driven tumor cell hyperproliferation in the combination group (Fig. 5F and G).

Given that TMPC-ANXA2 axis orchestrates initial bacterial adherence, we hypothesized that its blockade would reduce the intratumoral bacterial burden. Indeed, quantification of viable *S. anginosus* recovered from homogenized tumor tissues demonstrated that LCKLSL administration significantly reduced bacterial persistence within tumor tissues (Fig. 5H). This reduction was further confirmed by species-specific qPCR, which showed markedly lower intratumoral *S. anginosus* signals in the LCKLSL co-treatment group than in the *S. anginosus*-alone group (Fig. S4H). Consequently, this restricted bacterial burden translated into a robust attenuation of downstream oncogenic signaling. Tumors from LCKLSL co-treatment mice exhibited a marked downregulation of the core NF-κB-associated inflammatory gene signature (Fig. 5I and Fig. S4G), consistent with our *in vitro* observations. This suppressed inflammatory state within the tumor microenvironment was further corroborated by prominently diminished TNFα protein expression in immunohistochemical analyses (Fig. 5J and K). Collectively, these *in vivo* data demonstrate that targeting the TMPC-ANXA2 interface effectively limits intratumoral *S. anginosus* burden, mitigates tumor-promoting inflammation, and restrains NPC progression.

## DISCUSSION

Our study identifies *S. anginosus* as a functional driver of NPC progression, moving beyond descriptive microbiome associations to establish a mechanistically defined, targetable microbial determinant. We demonstrate that *S. anginosus* promotes tumor growth through the TMPC-ANXA2-mediated activation of the NF-κB pathway, and that disrupting this defined host-microbe interface restrains tumor progression *in vivo*. These findings shift the paradigm from viewing oral bacteria as passive bystanders to active contributors in the NPC tumor microenvironment.

The nasopharynx represents a unique anatomical junction, continuously bathed in saliva and oral secretions (22). Unlike other mucosal sites, such as the gastrointestinal tract, which is protected by formidable barriers like gastric acidity (23), the anatomical continuity between the oral cavity and nasopharynx facilitates sustained microbial translocation (20, 21, 24). Our findings suggest that under dysbiotic conditions, *S. anginosus* utilizes this geographical advantage to colonize the nasopharyngeal epithelium. Critically, the pro-inflammatory and pro-tumorigenic effects we observed, characterized by a robust NF-κB and TNFα transcriptional response, suggest that *S. anginosus* acts as an important contributor to a microbe-driven inflammatory niche. In the context of NPC, such sustained inflammatory signaling is known to recruit myeloid-derived suppressor cells (MDSCs) (25, 26) and tumor-associated neutrophils (27, 28), potentially shielding the tumor from immune surveillance.

Interestingly, while several *Streptococcus* species inhabit the NPC niche, *S. anginosus* exhibits a superior capacity for tumor promotion. Despite close phylogenetic relationships, these species display marked disparities in their ability to adhere to NPC cells and promote malignant phenotypes. Our comparative sequence analysis reveals that this functional dominance is rooted in the evolutionary conservation of specific TMPC residues. Minor structural variations at the TMPC-ANXA2 interaction interface dictate binding affinity, explaining why closely related species fail to trigger the same magnitude of oncogenic signaling. This pattern mirrors findings in other cancer-associated bacteria, such as the marked variability in pro-tumorigenic capacity among different *Fusobacterium nucleatum* clades (29–31). This molecular resolution highlights that bacterial pathogenicity in cancer is not merely a function of microbial abundance, but is determined by highly specific, evolved interaction modules.

A central finding of our study is the dual role of the host receptor ANXA2 (32, 33). While the TMPC-ANXA2 axis was recently shown to activate the MAPK pathway in gastric cancer (34), our results identify this interface as a potent driver of NF-κB signaling in NPC. This suggests that while the TMPC-ANXA2 interaction is a conserved molecular bridge across mucosal sites, it recruits distinct downstream oncogenic programs depending on the tissue context. We propose that ANXA2 not only facilitates bacterial adhesion but also functions as an intrinsic oncogenic driver that is frequently overexpressed in NPC. This creates a malignant synergy where host susceptibility (high ANXA2 expression) and microbial pathogenicity (*S. anginosus* persistence) converge. This convergence likely triggers a feed-forward loop, where bacterial-induced inflammation further stabilizes the pro-tumorigenic state. Our clinical data linking high ANXA2 levels with poorer patient survival reinforces the idea that this interface is a critical determinant of disease severity.

NPC is etiologically linked to EBV infection, and EBV-associated signaling may provide an important cellular context for microbial stimulation. EBV latent infection can promote inflammatory, survival, and immune-regulatory programs in NPC cells, including pathways converging on NF-κB signaling (35, 36). In the present study, we identify the TMPC-ANXA2 axis as a host-microbe interface through which *S. anginosus* activates NF-κB-related inflammatory signaling. These findings raise the possibility that *S. anginosus* and EBV may cooperate at the level of inflammatory signaling: EBV-positive epithelial cells may be more permissive to bacterial inflammatory stimulation, while *S. anginosus*-induced NF-κB activation may further reinforce an EBV-associated pro-tumorigenic state. Consistent with this possibility, our additional experiments in the EBV-positive C666-1 cell line showed that *S. anginosus* enhanced proliferative and migratory phenotypes in an EBV-positive NPC background (Fig. S5). Beyond shared inflammatory signaling, *S. anginosus*-induced cytokine and NF-κB activation may also influence EBV persistence, latency-associated gene expression, or viral reactivation dynamics, thereby contributing to a feed-forward interaction between microbial stimulation and EBV-associated oncogenic programs. This potential *S. anginosus*-EBV crosstalk provides an important direction for understanding how oral bacteria may interact with viral oncogenesis in NPC.

From a translational perspective, the TMPC-ANXA2 interface offers a compelling vulnerability for precision intervention. Current microbiome-based strategies often rely on broad-spectrum antibiotics, which risk inducing further dysbiosis and systemic resistance (37–40). By pharmacologically or competitively blocking the TMPC-ANXA2 attachment site, we demonstrate that it is possible to intercept the inflammatory fuel provided by the microbiome without disrupting the broader commensal balance. This non-antibiotic, targeted interference strategy represents a rational therapeutic avenue in microbiome-driven malignancies, moving from blunt antimicrobial force toward the targeted decoupling of specific pro-tumorigenic host-microbe interactions.

Several limitations remain. Our isolate-based experiments establish that *S. anginosus* can directly promote NPC-relevant phenotypes through the TMPC-ANXA2/NF-κB axis, but they do not fully reproduce the complexity of the native microbial community, where bacterial behavior may be influenced by co-occurring species, nutrient exchange, biofilm organization, and host environmental pressures. Previous work by Debelius et al. suggested that NPC-associated microbial alterations involve community-level changes, including co-occurring and co-excluding relationships among oral taxa, such as *Granulicatella*, *Streptococcus*, *Prevotella*, and *Veillonella*, rather than isolated shifts in a single organism (19). In line with this view, our *S. anginosus*-centered network analysis showed that *S. anginosus* was not retained in a comparable local association network in healthy controls but was linked to 34 direct neighboring taxa in NPC samples, including positive associations with oral species, such as *Streptococcus constellatus*, *Actinomyces oris*, *Prevotella dentalis*, and *Lancefieldella rimae* (Fig. S6). These findings suggest that *S. anginosus* is embedded within an NPC-associated microbial neighborhood that may shape, support, or reflect its biological activity. Thus, although our mechanistic experiments define a direct TMPC–ANXA2/NF-κB pathway for *S. anginosus*, they should be interpreted alongside broader community-level microbial restructuring. Future studies using defined microbial consortia, multi-species co-culture systems, community perturbation models, and longitudinal sampling will be needed to determine how *S. anginosus* functions within the broader NPC-associated microbial ecosystem.

In addition, the stage-associated analysis should be interpreted cautiously because only two patients in this cohort were classified as stage I; therefore, the observed increase in *Streptococcus* abundance across ordered tumor stages requires validation in larger cohorts with greater representation of early-stage disease. The *in vivo* model also has inherent limitations. Our subcutaneous xenograft model with intratumoral bacterial administration was designed to test whether local exposure to *S. anginosus* can promote tumor growth and inflammatory signaling *in vivo*. This model does not establish natural oral colonization, spontaneous bacterial engraftment, or the route by which oral bacteria may access the nasopharyngeal tumor microenvironment. Future orthotopic NPC models, longitudinal bacterial-tracking approaches, and microbial exposure systems will be required to define the dynamics of *S. anginosus* persistence and host-microbe interaction in a more physiologically relevant setting.

### Conclusions

In conclusion, we have established a definitive mechanistic link between *S. anginosus* enrichment and NPC progression. By characterizing the TMPC-ANXA2 based NF-κB inflammatory axis, we provide a crucial molecular framework for understanding oral-nasopharyngeal crosstalk and a rational roadmap for the development of targeted therapies to intercept bacteria-driven malignancies.

## MATERIALS AND METHODS

### Human participants

This study employed a multi-cohort design integrating an in-house discovery cohort and an independent external validation cohort to investigate oral microbiome alterations associated with NPC. The discovery cohort included 75 histopathologically confirmed NPC patients and 27 age-matched healthy controls (18–70 years) recruited from the First Affiliated Hospital of Sun Yat-Sen University. Exclusion criteria for both groups included the presence of systemic diseases (e.g., diabetes mellitus or cardiovascular disorders), antibiotic use within 2 weeks prior to sample collection, and incomplete clinical or demographic information. Saliva samples from this cohort were used for shotgun metagenomic sequencing.

An independent external validation cohort was derived from a previously published study (20), comprising 78 NPC patients and 38 healthy controls recruited from the Sun Yat-Sen University Cancer Center and the First Affiliated Hospital of Sun Yat-Sen University. Raw 16S rRNA gene sequencing data (PRJNA1011041) from this cohort were obtained and reanalyzed to validate taxonomic patterns identified in the discovery cohort.

In addition, an external intratumoral metagenomic cohort was included from a previously published study (12), comprising 44 NPC tissue samples collected at the Sun Yat-sen University Cancer Center. Raw sequencing data (PRJNA781360) were downloaded and reprocessed to examine intratumoral microbial composition within the NPC microenvironment and to assess the presence of candidate bacterial taxa identified in the saliva-based cohort.

### Animals

Female BALB/c nude mice (5 weeks old) were used for xenograft experiments. Mice were housed under specific pathogen-free (SPF) conditions with *ad libitum* access to food and water.

### Cell lines

The human nasopharyngeal carcinoma cell line SUNE-1 was used in this study and was kindly provided by Prof. Weihua Jia (Sun Yat-Sen University Cancer Center). The cell line was routinely tested and confirmed to be negative for mycoplasma contamination prior to use.

### Microorganisms

*S. anginosus*, *S. vestibularis*, *S. sanguinis*, *S. gordonii*, *S. parasanguinis*, and *S. salivarius* were isolated from saliva samples of patients with NPC and maintained as laboratory isolates. Species identity was confirmed by matrix-assisted laser desorption/ionization time-of-flight mass spectrometry (MALDI-TOF MS; Bruker MALDI Biotyper Smart) and validated by full-length 16S rRNA gene sequencing followed by NCBI nucleotide database alignment.

### Saliva collection and DNA extraction

Unstimulated saliva samples were collected from all participants within 30 min of morning waking, with participants abstaining from eating, drinking, smoking, and oral hygiene prior to collection. No patients had received radiotherapy at the time of saliva collection. Approximately 3 mL of saliva was collected into sterile tubes under standardized conditions, immediately transported to the laboratory on ice, and stored at −80°C until further processing.

Total genomic DNA was extracted from saliva samples using the HiPure Bacterial DNA Kit (Magen) according to the manufacturer’s instructions, with negative and positive controls processed in parallel. DNA concentration and purity were assessed using the Qubit dsDNA HS Assay Kit (Invitrogen), and DNA samples meeting quality requirements were used for downstream library construction.

### Shotgun metagenomic sequencing and analysis

High-quality genomic DNA from the in-house discovery cohort’s saliva samples was subjected to shotgun metagenomic sequencing. Paired-end libraries with an average insert size of ~350 bp were constructed using the Illumina TruSeq DNA PCR-Free Library Preparation Kit and sequenced on the Illumina NovaSeq platform using a paired-end 150-bp strategy.

Raw sequencing reads were quality-filtered using fastp (version 1.3.4) with default parameters. Host-derived reads were removed by alignment to the human reference genome (GRCh38/hg38) using Bowtie2 (version 2.4.0) with default parameters (41), and host-depleted paired reads were extracted using the --un-conc parameter. The remaining non-host reads were aligned to the Human Oral Microbiome Database (HOMD, version 4.2) reference genome collection using Bowtie2 (version 2.4.0) with default parameters. The resulting alignments were sorted and indexed using samtools (version 1.23.1). Taxon-level abundance profiles were generated based on raw read counts derived from alignment files, and reads were assigned to taxonomic ranks based on HOMD genome annotations. Species-level abundances were obtained by aggregating mapped reads according to the corresponding HOMD taxonomic annotations.

### 16S rRNA gene sequencing data processing

For the external validation cohort, raw 16S rRNA gene sequencing data were obtained from the previously published NPC oral microbiome study (20). In that study, the V4 hypervariable region of the 16S rRNA gene was amplified using barcode-labeled primers 515F (5′-GTGCCAGCMGCCGCGGTAA-3′) and 806R (5′-GGACTACHVGGGTWTCTAAT-3′), followed by sequencing on the Illumina MiSeq 2500 platform. After demultiplexing based on sample barcodes, sequence denoising was performed using the DADA2 pipeline implemented in QIIME 2 (version 2026.4) (42) to infer amplicon sequence variants (ASVs). In the present study, the resulting ASV sequences were taxonomically annotated against the HOMD database (version 16.03) using QIIME 2 with default parameters (confidence threshold = 0.7). ASVs assigned to the same genus were collapsed into genus-level abundance tables for validation of taxonomic patterns identified in the shotgun metagenomic analysis.

### Bacterial culture

All strains were routinely cultured on blood agar plates and propagated in brain heart infusion (BHI) broth at 37°C under anaerobic conditions. For functional assays, bacteria were grown to the mid-logarithmic phase, harvested by centrifugation, washed twice with sterile phosphate-buffered saline (PBS), and resuspended at the indicated density.

### Cell culture

Cells were maintained in RPMI-1640 medium supplemented with 10% fetal bovine serum (FBS) and 1% penicillin-streptomycin at 37°C in a humidified incubator with 5% CO₂. Cells were used within 15 passages after thawing and were routinely tested to confirm the absence of mycoplasma contamination.

### Co-culture of bacteria and cells

SUNE-1 (EBV-negative) and C666-1 (EBV-positive) cells were seeded and cultured to approximately 70%–80% confluence prior to bacterial exposure. *Streptococcus* species were prepared as aforementioned and resuspended in antibiotic-free RPMI-1640 medium with 10% FBS, then added to cells at a multiplicity of infection (MOI) of 1 and co-incubated for 6 h at 37°C in a 5% CO₂ humidified incubator. After co-incubation, cell monolayers were washed twice with warm PBS and replenished with fresh medium containing 1% penicillin-streptomycin. For inhibition experiments, the annexin A2-targeting hexapeptide competitive inhibitor LCKLSL (MCE, HY-P2333A) (43) was premixed with bacterial suspensions at the indicated concentrations and maintained throughout the co-incubation period.

### Cell proliferation assay

Cell proliferation was assessed using the Cell Counting Kit-8 assay (CCK-8; Biosharp). SUNE-1 cells were seeded into 96-well plates at a density of 3,000 cells per well and allowed to adhere for 24 h prior to bacterial co-culture. After co-incubation, cells were washed and cultured in fresh medium containing 1% penicillin-streptomycin. At the indicated time points (6, 24, and 42 h), CCK-8 reagent was added and incubated for 2 h at 37°C. Absorbance was measured at 450 nm using a microplate reader (Thermo Multiskan SkyHigh). Experiments were independently repeated at least three times.

### Wound healing assay

Cells were cultured to confluence in six-well plates, and a linear scratch was generated using a sterile 200-μL pipette tip. Cells were washed and exposed to *Streptococcus* species at an MOI of 1 for 6 h, followed by replacement with fresh medium containing 1% penicillin-streptomycin. For inhibition experiments, LCKLSL was included during the co-culture period at a final concentration of 50 μg/mL. Images were captured at 0, 6, and 24 h, and wound closure was quantified using ImageJ software.

### Bacterial adhesion assay

SUNE-1 cells were co-incubated with *S. anginosus* or *S. vestibularis* at an MOI of 1 for 2 h in antibiotic-free medium. After washing, cells were detached using 0.25% trypsin-EDTA. Cell-associated bacteria were serially diluted and plated on blood agar plates. CFUs were enumerated after incubation at 37°C for 24 h.

### Xenograft establishment and monitoring

Exponentially growing SUNE-1 cells were washed twice with sterile PBS, resuspended in a 1:1 Matrigel (Corning)/PBS mixture, and 8 × 10⁶ cells in 100 μL were subcutaneously injected into the right flank of each mouse. Tumor growth was measured every 2 days with digital calipers starting at 14 days post-implantation, and volume was calculated using the modified ellipsoid formula: *V* = 0.5 × length × width².

### Streptococcal administration and experimental design

When tumors reached an average volume of approximately 300 mm³, mice were randomized for intratumoral treatment. For comparative evaluation of streptococcal species, mice were divided into three groups: vehicle control (PBS), *S. vestibularis* (1 × 10⁵ CFU), and *S. anginosus* (1 × 10⁵ CFU). Treatments were administered by intratumoral injection in a total volume of 50 μL every 3 days.

For inhibition studies targeting the TMPC-ANXA2 interaction, mice were allocated to four treatment groups: vehicle control, inhibitor alone, *S. anginosus* alone, or *S. anginosus* combined with inhibitor. The ANXA2-targeting peptide inhibitor LCKLSL (5 μg per injection) was co-administered with *S. anginosus* in the same intratumoral injection volume. All treatments were performed at three-day intervals throughout the experimental period.

### Tumor harvest and sample allocation

At the experimental endpoint, mice were euthanized, and tumors were excised, photographed, and weighed. Tumor tissues were sectioned and allocated for downstream analyses, including histological evaluation, immunohistochemistry, RNA extraction, and bacterial quantification, according to the requirements of each assay.

### Quantification of intratumoral bacteria

To assess bacterial persistence within tumor tissues, approximately one-quarter of each excised tumor was weighed, mechanically homogenized in sterile PBS, and serially diluted. Homogenates were plated onto blood agar plates and anaerobically incubated at 37°C for 24 h. Colony-forming units (CFU) were enumerated and normalized to tumor weight to determine intratumoral bacterial burden (CFU/g). In addition, species-specific qPCR was performed to verify the presence and relative abundance of administered bacteria in tumor tissues (see “RT-qPCR analysis” below for details).

### RNA sequencing and transcriptomic analysis

Total RNA was extracted from SUNE-1 cells using the PureLink RNA Mini Kit (Thermo Fisher Scientific). RNA concentration was assessed using a NanoDrop spectrophotometer (Thermo NanoDrop Lite), and RNA integrity was evaluated using an Agilent Bioanalyzer; samples with RIN ≥ 7.0 were used for library construction. RNA-seq libraries were prepared using a poly(A) enrichment strategy and sequenced on an Illumina platform by BGI Genomics (Shenzhen, China), generating paired-end reads.

Reads were quality-checked using FastQC (version 0.11.9) (44) and trimmed using Trimmomatic (version 0.36) (45). Clean reads were aligned to the human reference genome (GRCh38) using STAR (46). Gene-level counts were generated using featureCounts (47). Differential expression analysis was conducted using DESeq2 (version 1.50.2) (48), with adjusted *P*-value < 0.05 considered statistically significant. Gene set enrichment analysis (GSEA) was performed using ClusterProfiler (version 4.18.2) (49) against hallmark and KEGG gene sets from MSigDB (v2023.2) (50). Normalized enrichment scores (NESs) and false discovery rates (FDRs) were used to assess pathway significance, and leading-edge genes were extracted for downstream visualization.

### RT-qPCR analysis

Total RNA was extracted from cultured cells or xenograft tumor tissues using the SteadyPure Universal RNA Extraction Kit (AG21017). RNA concentration and purity were assessed using a NanoDrop spectrophotometer. cDNA was synthesized from 1 μg total RNA using Evo M-MLV RT Premix for qPCR (AG11706). qPCR was performed using PowerUp SYBR Green Master Mix (Thermo Fisher Scientific) on a Bio-Rad CFX96 Real-Time PCR system. Primer sequences are provided in Table S1. Relative gene expression was calculated using the 2^−ΔΔ^*^Ct^* method and normalized to *GAPDH*. Each reaction was run in technical triplicate, and at least four independent biological replicates were included per condition.

### Co-immunoprecipitation and western blotting

Full-length ANXA2 and TMPC coding sequences were cloned into pUC57-ANXA2-HA and pUC57-TMPC-Myc vectors, respectively (Fig. S4B). HEK293T cells were cultured in DMEM supplemented with 10% FBS at 37°C under 5% CO₂ and transiently transfected with the indicated plasmids using Lipofectamine 3000 (Thermo, L3000001). After 48–72 h post-transfection, cells were harvested and lysed in NP-40 lysis buffer containing protease and phosphatase inhibitors (Beyotime, P0013). Protein concentrations were quantified using a BCA assay (Thermo, 23225), and equal amounts of lysates were used for immunoprecipitation.

For co-IP assays, cell lysates were incubated with anti-HA antibody (for ANXA2 pull-down) and protein A/G magnetic beads (Vazyme, PB201-02); IgG was used as a negative control. The immunocomplexes were washed, eluted in SDS loading buffer, and separated by SDS-PAGE, followed by transfer onto PVDF membranes. For Western blotting, membranes were blocked in 5% non-fat milk and incubated with primary antibodies against HA and Myc, followed by HRP-conjugated secondary antibodies. Signals were detected using enhanced chemiluminescence (ECL). Tubulin was used as a loading control for input samples. Antibody details are provided in Table S3.

### Prediction and sequence analysis of the TMPC-ANXA2 interaction

Computational prediction and comparative sequence analysis were performed to characterize the potential interaction between the streptococcal surface protein TMPC and the host membrane protein annexin A2 (ANXA2). Protein-protein interaction structures were predicted using AlphaFold 3 (51). For each interaction pair, multiple models were generated, and the top-ranked model was selected based on interface confidence metrics, including ipTM scores and predicted aligned error (PAE) profiles. The predicted complexes were visualized in PyMOL (version 3.1.0). The amino acid sequences of the proteins used in the study can be found in Table S2.

For comparative sequence analysis, TMPC homolog sequences from *S. anginosus*, *S. vestibularis*, *S. sanguinis*, *S. gordonii*, *S. parasanguinis*, and *S. salivarius* were aligned using ClustalW (52) with default parameters. Multiple sequence alignments were rendered using ESPript 3.0, and residues corresponding to predicted interface sites were mapped onto the alignment to summarize substitutions and indels across species.

### Immunohistochemistry and histology

Excised xenograft tumors were fixed in 10% neutral-buffered formalin for 24 h, embedded in paraffin, and sectioned at 4 μm. For histology, sections were deparaffinized, rehydrated, and stained with hematoxylin and eosin (H&E).

For immunohistochemistry (IHC), sections underwent heat-induced antigen retrieval in citrate buffer (pH 6.0), followed by blocking of endogenous peroxidase activity and nonspecific binding. Sections were incubated overnight at 4°C with primary antibodies against Ki67 (1:200; Servicebio, Cat# GB121141) or TNFα (1:100; Boster, Cat# BA0131), followed by appropriate secondary antibodies and visualization using a DAB detection system. Slides were counterstained with hematoxylin, dehydrated, and mounted. Two independent pathologists assessed staining intensity and the proportion of positive cells using the immunoreactive score (IRS) method (53).

### Statistical analysis

Differences in overall microbial community structure were assessed using PERMANOVA based on Bray-Curtis dissimilarity and visualized by principal coordinate analysis (PCoA). Differentially abundant taxa were primarily identified using ALDEx2, with ANCOM, ANCOM-BC2, and LEfSe used as complementary analyses to assess the robustness of differential signatures. Two-group comparisons were performed using the Wilcoxon rank-sum test, and multi-group comparisons were performed using one-way ANOVA (with Tukey’s HSD) for normally distributed data, or the Kruskal-Wallis test (with Dunn’s test) for non-normally distributed data. Monotonic trends across ordinal tumor stages (T1–T4) were assessed using the Jonckheere-Terpstra test. The survival differences were evaluated with the Kaplan-Meier method and log-rank test. Species-level microbial association networks were constructed using Spearman correlations based on CLR-transformed relative abundance profiles to explore the community context surrounding *S. anginosus*. All statistical analyses were conducted in R (version 4.3.2). Unless otherwise stated, data are presented as mean ± SD, and a *P*-value < 0.05 was considered statistically significant.

## Data Availability

A STORMS (Strengthening The Organizing and Reporting of Microbiome Studies) checklist is available at https://doi.org/10.5281/zenodo.19204351. The metagenomic shotgun sequencing data generated in this study have been deposited in the NCBI BioProject database under accession number PRJNA1475086. The RNA sequencing data are available in the NCBI BioProject database under accession number PRJNA1393446. All data supporting the findings of this study are publicly available from the indicated repositories. The code developed for data analysis is available at https://github.com/bmryggdrasil/2025-NPC-project-Analysis.

## AFTER EPUB

*[This article was published on 25 August 2026 with an error in Acknowledgments. The Acknowledgments were corrected on 11 September 2026 and in the current version, posted on 14 September 2026.]*

## SUPPLEMENTAL MATERIAL

Supplemental material msystems.00357-26-s0001.docx Fig. S1-S6; Tables S1-S3.

ASM does not own the copyrights to Supplemental Material that may be linked to, or accessed through, an article. The authors have granted ASM a non-exclusive, world-wide license to publish the Supplemental Material files. Please contact the corresponding author directly for reuse.
