# Supplementary material for: Integrative microbiome and functional profiling identify Streptococcus anginosus as a pro-tumorigenic driver in nasopharyngeal carcinoma
Source: mSystems. 2026 Aug 25;11(9):e00357-26. doi: 10.1128/msystems.00357-26 (PMC13595960; doi:10.1128/msystems.00357-26)
Supplement: Supplementary file 1 [file msystems.00357-26-s0001.docx]

**Integrative microbiome and functional profiling identify *Streptococcus anginosus* as a pro-tumorigenic driver in nasopharyngeal carcinoma**

**Authors and Affiliations:**

Yifan Zhu^1,2†^, Lewei Zhou^1,2†^, Chenyu Zhang^1,2^, Zonglin Li^1,2^, Tao Ding^1,2 #^, Ying Su^1,2,a #^

^1^ Department of Immunology and Microbiology, Zhongshan School of Medicine, Sun Yat-sen University, Guangzhou 510080, China

^2^ Key Laboratory of Human Microbiome and Chronic Diseases (Sun Yat-sen University), Ministry of Education, Guangzhou 510080, China

**Running title:** *S. anginosus* drives NPC tumorigenesis

^a^ **ORCID**: 0009-0004-1566-603X

**^#^ Corresponding authors:**

Ying Su, Ph.D.

Tao Ding, Ph.D.

Zhongshan School of Medicine, Sun Yat-sen University, Guangzhou 510080, China

Fax: 86-20-87333380

Email: suying8@mail.sysu.edu.cn; dingt8@mail.sysu.edu.cn

† These authors contributed equally to this work. Author order was determined in order of increasing seniority.

**Fig. S1: Robustness and validation of differentially abundant oral bacterial genera in NPC.**

Differentially abundant bacterial genera between NPC patients (n = 75) and healthy controls (n = 27) in the discovery cohort identified using three complementary compositional methods: **(A)** ANCOM, **(B)** ANCOM-BC2, and **(C)** LEfSe. Bars represent the signed W statistic (ANCOM), log fold-change (ANCOM-BC2), or LDA score (LEfSe). Genera highlighted in red (*Actinomyces*, *Streptococcus*, and *Granulicatella*) were consistently enriched in NPC across all methods, indicating robust associations. **(D)** Relative abundance of *Actinomyces* and *Granulicatella* across individual saliva samples in the external validation cohort (NPC, n = 78; controls, n = 38). *Actinomyces* and *Granulicatella* did not differ significantly between groups. Relative abundances are shown on a log10-transformed scale. Boxes indicate the median and interquartile range, and whiskers represent 1.5× the interquartile range. Statistical significance was assessed using two-sided Wilcoxon rank-sum tests; NS, not significant. **(E)** Mean relative abundance and prevalence of the top bacterial species detected in NPC tumor tissue metagenomic samples (PRJNA781360). The upper bar plot shows species-level relative abundance, with bars colored by genus. The lower heat strip shows prevalence across tumor tissue samples. Species outside the dominant genera are grouped as “Other”. Prevalence is shown as the proportion of positive samples.

**Fig. S2: Quantitative and morphological validation of differential proliferative and migratory effects among oral Streptococcus species.**

**(A)** Cell growth rate coefficients (β) derived from time-course CCK-8 proliferation assays of SUNE-1 nasopharyngeal carcinoma cells co-cultured with six representative *Streptococcus* species isolated from NPC saliva. Growth rates were estimated by linear regression of normalized absorbance values over the exponential growth phase. Several species, including *S. anginosus*, *S. sanguinis*, *S. gordonii*, and *S. parasanguinis*, exhibited significantly higher growth rate coefficients compared with control conditions, whereas *S. salivarius* and *S. vestibularis* showed minimal or no stimulatory effects. Data are presented as mean ± SD from *n* = 6 independent biological replicates per condition. **(B)** Representative phase-contrast images of scratch-wound assays performed on SUNE-1 cells co-cultured with individual *Streptococcus* species at 0 h and 24 h (*n* = 6). Images illustrate differential wound closure dynamics across species, with *S. anginosus* inducing the most pronounced acceleration of wound closure. Quantitative analysis of wound closure is shown in Fig. 2C. Statistical significance in (**A**) was determined using one-way ANOVA followed by Tukey’s HSD test compared to the Ctrl group. ***P* < 0.01; ns, not significant.

**Fig. S3: Lack of TNFα–NF-κB pathway enrichment in SUNE-1 cells exposed to *S. vestibularis.***

**(A)** Gene set enrichment analysis (GSEA) of the TNFα signaling via NF-κB pathway in SUNE-1 cells following exposure to *S. vestibularis*. The enrichment plot shows the running enrichment score across the ranked gene expression dataset. Representative genes (*RELB*, *VEGFA*, *JUN*, and *CXCL2*) are indicated. In contrast to *S. anginosus*-treated cells (see Fig. 3B), *S. vestibularis* exposure failed to induce significant enrichment, as indicated by a low normalized enrichment score (NES = 0.74) and lack of statistical significance (*P* = 0.99). **(B, C)** Quantitative PCR analysis of NF-κB-associated and EMT-related genes (*RELB*, *JUN*, and *VIM*) in SUNE-1 cells (*in vitro*, **B**) and in xenograft tumor tissues (*in vivo*, **C**) following treatment with the indicated *Streptococcus* species (n = 4 per group). *S. vestibularis* showed no significant stimulatory effect compared to the control group, whereas *S. anginosus* induced robust gene expression. **(D)** Fold change of intratumoral *Streptococcus* abundance in xenograft tumors following intratumoral administration of *S. anginosus* or *S. vestibularis* (n = 4 per group). Data in **(B),** **(C)** and **(D**) are presented as mean ± SD. Statistical significance was determined using one-way ANOVA followed by Tukey’s HSD test. **P* < 0.05; ***P* < 0.01; ****P* < 0.001; ns, not significant.

**Fig. S4: Structural and functional validation of the TMPC-ANXA2 interaction and LCKLSL-mediated inhibition.**

**(A)** Predicted structural model of the TMPC-ANXA2 complex generated using AlphaFold 3. TMPC from *S. anginosus* is shown in gold and ANXA2 in blue. Insets show enlarged views of the predicted interaction interface, with representative TMPC residues contributing to host binding highlighted. **(B)** Schematic illustration of plasmid constructs used for co-immunoprecipitation assays. **(C)** Representative raw Western blot (WB) and immunoprecipitation (IP) images showing TMPC-ANXA2 interaction in transfected cells. **(D)** Growth curves of six representative oral *Streptococcus* species cultured in the presence of the ANXA2-targeting peptide inhibitor LCKLSL or vehicle control (PBS). Bacterial growth was monitored over 12 h under standard conditions. LCKLSL treatment did not alter the growth kinetics of any tested species (n = 3 per condition). **(E)** Representative phase-contrast images of scratch-wound assays performed on SUNE-1 cells at 0 h and 24 h under the indicated treatment conditions. LCKLSL alone did not affect basal wound closure, whereas it markedly attenuated the *S. anginosus*-induced migratory response. Quantitative analysis is provided in Fig. 4E. **(F, G)** Quantitative PCR analysis of NF-κB-associated and EMT-related genes (*RELB*, *JUN*, and *VIM*) in SUNE-1 cells (*in vitro*, **F**) and in xenograft tumor tissues (*in vivo*, G) following treatment with *S. anginosus* in the presence or absence of LCKLSL (n = 4 per group). LCKLSL significantly suppressed *S. anginosus*-induced upregulation of these genes but had no effect on their basal expression levels. **(H)** Fold change of intratumoral *S. anginosus* abundance in xenograft tumors following intratumoral administration of *S. anginosus* with or without LCKLSL (n = 4 per group). *S. anginosus* was abundant in tumors after bacterial treatment, whereas co-administration with LCKLSL significantly reduced bacterial levels. Data in **(F)**, **(G)** and **(H)** are presented as mean ± SD. Statistical significance was determined using one-way ANOVA followed by Tukey’s HSD test (**F**) and **(H)**, or Welch’s ANOVA followed by the Games-Howell *post hoc* test (**G**). **P* < 0.05; ***P* < 0.01; ****P* < 0.001; ns, not significant.

**Fig. S5: Functional validation of *S. anginosus*-induced proliferative and migratory phenotypes in EBV-positive C666-1 cells**. **(A)** Proliferation of EBV-positive C666-1 cells following exposure to six representative oral *Streptococcus* species, measured by CCK-8 assay over 42 h. **(B)** Quantification of the cell growth rate coefficient derived from the proliferation curves in **(A)**. **(C)** Quantification of wound healing in C666-1 cells following exposure to the indicated *Streptococcus* species. **(D)** Representative phase-contrast images of scratch-wound assays at 0 h and 24 h corresponding to **(C)**. **(E)** C666-1 cell viability at 24 h following treatment with *S. anginosus* in the presence or absence of LCKLSL at the indicated concentrations. **(F)** Quantification of wound healing in C666-1 cells treated with *S. anginosus* with or without LCKLSL. **(G)** Representative phase-contrast images of scratch-wound assays corresponding to **(F)**. Data are presented as mean ± SD. Statistical significance was determined using one-way ANOVA followed by Tukey’s HSD test for multiple-group comparisons unless otherwise indicated. * *P* < 0.05; ***P* < 0.01; ****P* < 0.001; ns, not significant.

**Fig. S6**: ***S. anginosus*-centered local microbial association network in NPC samples.** The network shows the local association neighborhood surrounding *S. anginosus* in NPC samples. Edges represent significant microbial associations retained at |Spearman ρ| ≥ 0.25 and FDR-adjusted P ≤ 0.05. Nodes represent bacterial species, and node size indicates mean relative abundance. Node colour indicates network modules identified by community detection. Orange solid edges indicate positive associations, whereas blue dashed edges indicate negative associations; edge width represents |Spearman ρ|. White-centered nodes indicate labelled key taxa, including *S. anginosus* and the top 10 taxa directly associated with *S. anginosus* ranked by association strength.

**Table S1. qRT-PCR primers used in this study.**

| **Gene** | **Primer** | **Sequence (5’ to 3’)** |
| --- | --- | --- |
| *GAPDH* | forward | AATCCCATCACCATCTTCC |
|  | reverse | TGAGGCTGTTGTCATACTTCT |
| *TNF* | forward | CCTCTCTCTAATCAGCCCTCTG |
|  | reverse | GAGGACCTGGGAGTAGATGAG |
| *NFKB1* | forward | GAAGCACGAATGACAGAGGC |
|  | reverse | GCTTGGCGGATTAGCTCTTTT |
| *RELB* | forward | CAGCCTCGTGGGGAAAGAC |
|  | reverse | GCCCAGGTTGTTAAAACTGTGC |
| *CXCL2* | forward | AAAGCTTGTCTCAACCCCG |
|  | reverse | GGTCAGTTGGATTTGCCATTTT |
| *VEGFA* | forward | AGGGCAGAATCATCACGAAGT |
|  | reverse | AGGGTCTCGATTGGATGGCA |
| *JUN* | forward | TCCAAGTGCCGAAAAAGGAAG |
|  | reverse | CGAGTTCTGAGCTTTCAAGGT |
| *CDH2* | forward | TGCGGTACAGTGTAACTGGG |
|  | reverse | GAAACCGGGCTATCTGCTCG |
| *VIM* | forward | AGTCCACTGAGTACCGGAGAC |
|  | reverse | CATTTCACGCATCTGGCGTTC |
| *SNAI1* | forward | TCGGAAGCCTAACTACAGCGA |
|  | reverse | AGATGAGCATTGGCAGCGAG |
| *S. anginosus* | forward | ATGAGTAAGCTGCTGCTGCTG |
|  | reverse | TTAGTCGTCGTCGTCGTCGTC |
| *S. vestibularis* | forward | GCTGAAGGTTCGCTGAAGGT |
|  | reverse | CCACTTGCTGCTGCTGTTCT |

**Table S2. Amino acid sequences of TMPC and Annexin A2 used in the study.**

| **Protein** | **Sequence** |
| --- | --- |
| Annexin A2 | MSTVHEILCKLSLEGDHSTPPSAYGSVKAYTNFDAERDALNIETAIKTKGVDEVTIVNILTNRSNAQRQD  IAFAYQRRTKKELASALKSALSGHLETVILGLLKTPAQYDASELKASMKGLGTDEDSLIEIICSRTNQEL  QEINRVYKEMYKTDLEKDIISDTSGDFRKLMVALAKGRRAEDGSVIDYELIDQDARDLYDAGVKRKGTDV  PKWISIMTERSVPHLQKVFDRYKSYSPYDMLESIRKEVKGDLENAFLNLVQCIQNKPLYFADRLYDSMKG  KGTRDKVLIRIMVSRSEVDMLKIRSEFKRKYGKSLYYYIQQDTKGDYQKALLYLCGGDD |
| Membrane lipoprotein TMPC | MNKKQWLGLGLVAVAAIGLAACGNRASKSDNKDAKTDLKAAIVTDTGGVDDKSFNQSAWEGLQAWGKENGLTKDHGFTYFQSTNESEYATNLEQAASSNYKLVFGIGFALRNAVESAAKDHSDINYVIIDDEIKGQKNVASALFADNEAAYLAGVAAAKTTKTKMIGFVGGIKGAVLTRFEKGFEAGVKSVDPSIKIDVKYAESFGDASKGKTIAAAQYAAGADVVYQVAGGTGAGVFNEAKSINETRNESDKVWVIGVDRDQNDEGKYKSKDGKESNFVLASSLKKVGGTVKDIANKTAKDKFPGGKTTTYGLKDGGVDLTTKNLSDDAKKAVEDAKAKILDGSIKVPTE |

**Table S3. Antibodies used in the study.**

| **Antibody** | **Supplier** | **Model** | **Dilution** |
| --- | --- | --- | --- |
| Myc | GenScript | A00704-100 | 1:500 |
| Goat Anti-mouse IgG | Beyotime | A0216 | 1:5000 |
| Tubulin | Bioworld | BS1482M | 1:5000 |
| HA | Vazyme | RA1004 | 1:2500 |
